# 11S Glycinin Up-Regulated NLRP-3-Induced Pyroptosis by Triggering Reactive Oxygen Species in Porcine Intestinal Epithelial Cells

**DOI:** 10.3389/fvets.2022.890978

**Published:** 2022-06-15

**Authors:** Lei Wang, Zhifeng Sun, Weina Xie, Chenglu Peng, Hongyan Ding, Yu Li, Shibin Feng, Xichun Wang, Chang Zhao, Jinjie Wu

**Affiliations:** ^1^College of Animal Science and Technology, Anhui Agricultural University, Hefei, China; ^2^Department of Food Science and Engineering, School of Agriculture and Biology, Shanghai Jiao Tong University, Shanghai, China

**Keywords:** 11S glycinin, allergy, ROS, NLRP-3, lentiviral transfection, IPEC-J2 cell, pyroptosis

## Abstract

11S glycinin is a major soybean antigenic protein, which induces human and animal allergies. It has been reported to induce intestinal porcine epithelial (IPEC-J2) cell apoptosis, but the role of pyroptosis in 11S glycinin allergies remains unknown. In this study, IPEC-J2 cells were used as an *in vitro* physiological model to explore the mechanism of 11S glycinin-induced pyroptosis. The cells were incubated with 0, 1, 5, and 10 mg·ml^−1^ 11S glycinin for 24 h. Our results revealed that 11S glycinin significantly inhibited cell proliferation, induced DNA damage, generated active oxygen, decreased mitochondrial membrane potential, and increased the NOD-like receptor protein 3 (NLRP-3) expression of IPEC-J2 cells in a dose-dependent manner. Further, IPEC-J2 cells were transfected with designed sh-NLRP-3 lentivirus to silence *NLRP-3*. The results showed that 11S glycinin up-regulated the silenced *NLRP-3* gene and increased the expression levels of apoptosis-related spot-like protein (ASC), caspase-1, the cleaved gasdermin D, and interleukin-1β. The IPEC-J2 cells showed pyrolysis morphology. Moreover, we revealed that N-acetyl-L-cysteine can significantly inhibit the production of reactive oxygen species and reduce the expression levels of NLRP-3 and the cleaved gasdermin D. Taken together, 11S glycinin up-regulated NLRP-3-induced pyroptosis by triggering reactive oxygen species in IPEC-J2 cells.

## Introduction

Soybean is an important economic crop and is the main source of plant protein in food, feed, and other industries (1). Soybean is one of the eight allergic foods that can affect the respiratory system (2), digestive system (3, 4), and seriously affect animal growth and health (5). 11S glycinin is the main antigenic component of soybean protein. It is a hexamer protein consisting of different subunits, each of which consists of an acidic peptide chain and a basic peptide chain. The structure is very stable and is not easily damaged in its natural state. After eating 11S soybean protein, an intestinal immune response is triggered in young animals, damaging the intestinal mucosal barrier, resulting in growth retardation, dyspepsia, and allergic diarrhea (6, 7).

The intestinal epithelial cell (IEC) layer is exposed to the intestinal lumen contents, participates in the selective absorption of nutrients, and acts as a barrier to the passive paracellular permeability of the intestinal lumen contents through the expression of a tight-junction between adjacent IECs. Soybean allergy usually induces intestinal inflammatory diseases, characterized by the atrophy and proliferation of crypt villi, which accelerate the apoptosis and migration of intestinal cells (8, 9). Our previous studies have determined that 11S glycinin decreases intestinal porcine epithelial (IPEC-J2) cell membrane potential (10), damages the cell cytoskeleton and cell tight junctions, and induces IPEC-J2 cell apoptosis *via* the p38/JNK/NF–κB signaling pathway (11). Abnormal activation of the MAPK and NF–κB signaling pathways promotes an inflammatory response, leading to inflammatory damage of the small intestine tissue of piglets (12). However, the inflammatory response is a complex process and is the result of the interaction and mutual regulation of multiple pathways. In the recent years, a large number of studies have revealed that epithelial pyroptosis plays a key role in allergic diseases (13, 14). Pyroptosis is a pro-inflammatory form of programmed cell death (PCD), which is characterized by the continuous expansion of cells until the rupture of the cell membrane, resulting in the release of cellular contents and then activating a strong inflammatory response (15, 16). NOD-like receptor protein 3 (NLRP-3) is a protein complex in cells. Its main function is to activate caspase-1, activated caspase-1 cleaves gasdermin D (GSDMD), induces pyroptosis and promotes the secretion of interleukin-1β (IL-1β), causing an inflammatory response (17, 18).

In our previous studies, it was often found that some IPEC-J2 cells were swollen, the cell membrane was ruptured, there were holes in the cell membrane, the cytoplasm flowed out, and the cell morphology was highly redolent of pyrolysis. However, whether cell pyroptosis exists in 11S glycinin-induced IPEC-J2 cell injury and the molecular mechanism thereof, has not been reported. Therefore, in this study, we aim to explore the underlying molecular mechanisms of 11S glycinin in inducing IPEC-J2 cells pyroptosis.

## Materials and Methods

### Chemicals and Reagents

11S glycinin was provided by Professor Shuntang Guo of China Agricultural University (patent number 200 410 029 589.4, China). The fetal bovine serum (FBS) was provided by Clark Bioscience (Richmond, VA, USA). The RPMI 1,640 medium was obtained from Thermo Fisher Scientific, Waltham, MA, USA. The cell Counting Kit-8 (CCK-8) was provided by Achieve Perfection Explore Biotechnology (Houston, USA). The LIVE/DEAD Cell Imaging Kit and Terminal Deoxynucleotidyl Transferase-mediated dUTP-biotin Nick end Labeling (TUNEL) Assay Kit were bought from Beyotime Biotechnology (Shanghai, China). 4,6- Benzamidine-2-phenylindole (DAPI) was obtained from Abcam (Cambridge, UK).

### Cell Culture

The IPEC-J2 cell lines were purchased from the China Center for Type Culture Collection (Wuhan, China) and cultured with RPMI 1,640 containing 10% FBS, 1% penicillin, and 1% streptomycin at 37 °C with 5% CO_2_ in a humidified atmosphere.

### Cell Viability Assay

IPEC-J2 cells were seeded into sterile 96-well plates at a density of 5 × 10^3^ cells per well. After a 24 h stabilization period, the cells were treated with different concentrations (0, 1, 5, and 10 mg·ml^−1^) of 11S glycinin for 24 h. After stimulation, CCK-8 regent was added followed by incubation at 37 °C in a 5% CO_2_ humidified atmosphere for 1 h. The absorbance was determined at 450 nm using a Multiskan MS plate reader (Thermo Fisher Scientific, Waltham, MA, USA). The ratio of viability of control cells was taken as 100%.

### Visual Fluorescence Effect of Live and Dead Cells

IPEC-J2 cells were seeded into sterile-glass-bottomed dishes at a density of 1.6 × 10^4^ cells/dish and cultured for 24 h. The cells were treated with different concentrations (0, 1, 5, and 10 mg·ml^−1^) of 11S glycinin. After 24 h stimulation, the cells were stained by the LIVE/DEAD Cell Imaging Kit, as per the manufacturer's instructions. The living and dead cells were observed and photographed using a JEM-1230 transmission electron microscope (JEOL, Akishima, Tokyo, Japan).

### Cellular ROS Detection

IPEC-J2 cells were seeded into sterile-6-well-culture plates at a density of 1.2 × 10^5^ cells/hole and cultured for 24 h. The cells were treated with different concentrations (0, 1, 5, and 10 mg·ml^−1^) of 11S glycinin. After 24 h stimulation, all the cells were treated with 10 μM of DCFH-DA (Elabscience Biotechnology, Wuhan, China) for 30 min, and the cells were observed and photographed using a fluorescent microscope (Olympus, Japan). The signal of cellular ROS was evaluated by flow cytometry (BD Biosciences, Franklin Lakes, NJ, USA), and the data were statistically analyzed by FlowJo software (Ashland, OR, USA).

### Flow Cytometric Analysis of JC-1 Staining

IPEC-J2 cells were seeded into sterile-6-well-culture plates at a density of 1.2 × 10^5^ cells/hole and cultured for 24 h. The cells were treated with different concentrations (0, 1, 5, and 10 mg·ml^−1^) of 11S glycinin. After 24 h stimulation, 500 μL ^−1^ × JC-1 staining solution and 500 μL DMEM solution were used to stain the cells for 30 min. The signal of cellular JC-1 was detected by flow cytometry (BD Biosciences, Franklin Lakes, NJ, USA).

### Terminal Deoxynucleotidyl Transferase-Mediated dUTP-Biotin Nick End Labeling Assay (TUNEL)

IPEC-J2 cells were seeded into sterile 24-well plates at a density of 8 × 10^4^ cells per hole for 24 h. The cells were incubated in different concentrations (0, 1, 5, and 10 mg·ml^−1^) of 11S glycinin for 24 h. The mode of cell death was determined according to the instructions of the deoxynucleotide terminal transferase-mediated nick TUNEL assay kit. The TUNEL-positive cells were marked with red fluorescence, and the nuclei were stained with 4′,6-diamidino-2-phenylindole (DAPI, Abcam, Cambridge, UK). The images were observed and collected under a fluorescence microscope.

### Immunofluorescence Assay

The IPEC-J2 cells were seeded at a density of 8 × 10^4^ cells per well into sterile 24-well plates covered with 14 mm cell slides and incubated with 1, 5, and 10 mg·ml^−1^ 11S glycinin for 24 h. The supernatant was discarded and the cells were washed three times in phosphate-buffered saline (PBS, 5 min/time). Polyoxymethylene (4%) was used to fix cells for 30 min and PBS was used to wash cells (3 times, 5 min/time). Triton X-100 (0.5%) was used to permeate the cell membrane for 15 min. The reaction was blocked with BCA (0.5%, 1 h). The IPEC-J2 cells were cultured overnight at 4°C with primary antibodies NLRP-3, after washing three times with PBS for 5 min each time. The FITC-bound secondary antibody (dilution of 1:100, E-AB-1016) was added and the cells were incubated for 1 h, and then washed with PBS (3 times, 5 min/time). After 10 min of nuclei staining with DAPI, cells were washed three times for 5 min each time with PBS. The sections were sealed with anhydrous glycerol and imaged under a confocal microscope (Olympus, Tokyo, Japan).

### Western Blotting

IPEC-J2 cells in logarithmic growth phase were seeded in sterile 6-well plates at a density of 1.6 × 10^5^ cells/ml for 24 h, and then treated with 0, 1, 5, and 10 mg·ml^−1^ 11S for 24 h, respectively. Samples were collected and IPEC-J2 cells were lysed with radioimmunoprecipitation assay (RIPA), lysis buffer (Beyotime Biotechnology, Shanghai, China), centrifuged (12,000 × g for 15 min at 4 °C), and the supernatant was collected. The Bicinchoninic acid (BCA) protein assay kit (Beyotime Biotechnology, Shanghai, China) was used to determine the protein concentration. The protein content of samples was adjusted to 40 μg and then diluted with 5 × loading buffer. The protein of samples was separated by 5% stacking gel and 10–15% separating gel and transferred to polyvinylidene fluoride (PVDF) membranes. The membranes were blocked with bovine serum albumin (BSA) for 4 h, washed 4 times with TBST, and then incubated with the indicated primary antibodies overnight at 4 °C. After washing with TBST, they were incubated with goat anti-rabbit immunoglobulin G (IgG) secondary antibody (1:5,000, Zen Bioscience, Chengdu, China) at room temperature for 1 h. After washing with TBST again, the pictures were collected by gel imager. The gray value of protein bands was analyzed using the ImageJ software.

### Lentiviral Transfection of IPEC-J2 Cells

Lentivirus (LV) recombinant expression *NLRP-3* and short hairpin RNA (shRNA) were synthesized using the GeneChem (Guangzhou, China). IPEC-J2 cells were transfected with designed sh-NLRP-3 lentivirus to silence *NLRP-3* (multiplicity of infection, MOI = 100). Transfection efficiency was observed using quantitative real-time polymerase chain reaction (qRT-PCR) and the western blot analysis. According to the results of the transfection efficiency, the cells were randomly divided into four groups. Control group (untreated), negative control (NC) group (transfected with empty vector lentivirus), sh-NLRP-3 group (transfected with designed sh-NLRP-3 lentivirus), sh-NLRP-3+10 mg·ml^−1^ 11S group (transfected with designed shNLRP3 lentivirus, then added 10 mg·ml^−1^ 11S glycinin and cultured for 24 h).

### qRT-PCR Assay

The mRNA expressions were detected using the qRT-PCR assay. Briefly, after IPEC-J2 cells were transfected by LV and incubated with 11S, samples were collected. Total RNA of samples were isolated and reverse transcribed into cDNA. The primer sequences used in qRT-PCR were shown in Table 1. The qRT-PCR detection method steps were performed as described previously (10).

**Table 1:** Primer sequences for qRT-PCR amplification.

**Gene**	**Forward primer (5^′^→3^**′**^)**	**Reverse primer (5^′^→3^′^)**	**product (bp)**
*NLPR3*	GACCTCAGCCAAGATGCAAG	TGCCCAGTCCAACATGATCT	163
*ASC*	ATCGACCTCACTGACAAGCT	TCAGGGGAAGGGCTTTGATT	169
*IL-1β*	TCTCTCACCCCTTCTCCTCA	CAGACACTGCTGCTTCTTGG	182
*Caspase-1*	CAGCCCCTCAGACAGTACAA	GCAGATTATGAGGGCAAGGC	173
*GSDMD*	TTCATGGTTCTGGAGACCCC	TCATGGAAGTAGAAGGGGCC	114
*GAPDH*	TGACCCCTTCATTGACCTCC	CCATTTGATGTTGGCGGGAT	160

### Transmission Electron Microscopy

After the samples were transfected and treated according to the method in Lentiviral transfection of IPEC-J2 cells, the supernatant was discarded after centrifugation at 1,000 rpm for 5 min. Then, 1 ml of 4% paraformaldehyde was added to each tube and fixed at 4°C for 12 h. The PBS was washed three times (4°C, 5,000 × *g*, 15 min) and then fixed with 2% osmotic acid for 4 h. After rinsing with PBS, gradient dehydration with 30–100% alcohol, osmotic embedding, cutting, and staining of ultra-thin sections were carried out. Photographs were clicked with a transmission electron microscope (JEM-1230, JEOL, Akishima, Tokyo, Japan).

### Enzyme-Linked Immunosorbent Assay

IPEC-J2 cells were seeded into sterile 6-well plates at a density of 1.6 × 10^5^ cells per well and transfected and treated according to the method described in Lentiviral transfection of IPEC-J2 cells. Subsequently, 500 μL of 0.1 mol/L Tris-HCl was added to each sample (pH 7.4, Beyotime Biotechnology, Shanghai, China), and the samples were sonicated. The samples were centrifuged for 10–15 min (1,000 rpm, 4°C) and the supernatant was collected. The cell lysates were collected, centrifuged at 1,000 rpm for 10 min, and the supernatant was aspirated. Caspase-1 and IL-1β content was assayed according to the ELISA kit instructions.

### Fluorescence Detection of Mitochondrial Membrane Potential

Mitochondrial membrane potential (MMP) was assayed using the Mitochondrial Membrane Potential and Apoptosis Detection Kit with MitoTracker Red CMXRos and Annexin V-FITC (Beyotime Biotechnology, Shanghai, China). IPEC-J2 cells were seeded into sterile 6-well plates at a density of 1.6 × 10^5^ cells per well and transfected and treated according to the method discussed in Lentiviral transfection of IPEC-J2 cells, following the instructions of the kit and observing under a fluorescence microscope (Olympus, Tokyo, Japan).

### Data Analysis and Processing

The experimental data are expressed as mean ± SD. ANOVA of SPSS 17.0 software was used for variance analysis, and the LSD method was used for significant comparison, with *P* < 0.05 indicating significant difference and *P* < 0.01 indicating extremely significant difference. Graph Pad Prism 7.0 software was used to draw the histogram.

## Results

### 11S Glycinin Suppressed IPEC-J2 Cells

**Figure 1:**
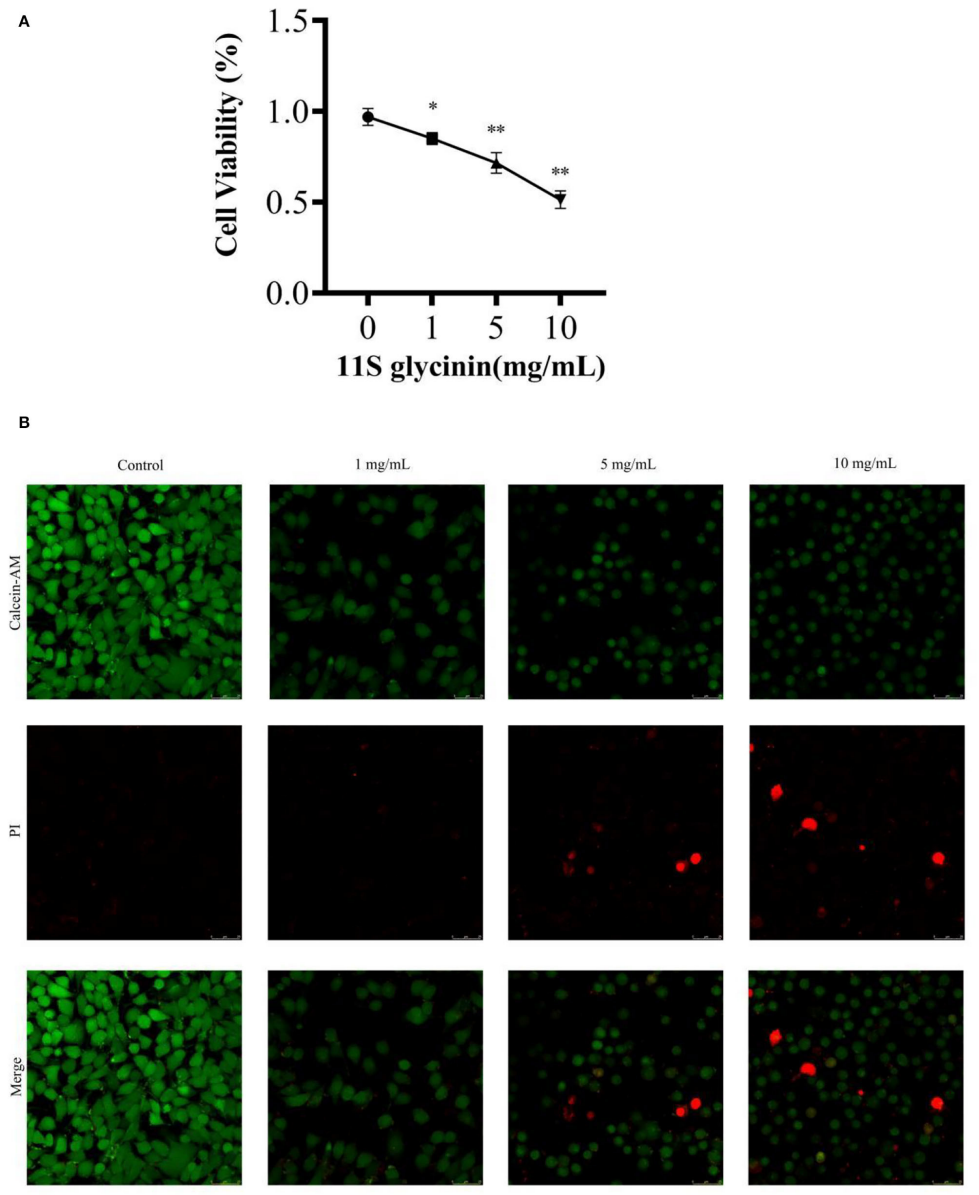
11S glycinin suppressed IPEC-J2 cells. **(A)** IPEC-J2 cells were stimulated with different concentrations (0, 1, 5, and 10 mg·ml^−1^) of 11S glycinin for 24 h, and cell viability was determined using the CCK-8 assay, *n* = 4. **(B)** Fluorescence photographs of IPEC-J2 cells stained by using the LIVE/DEAD™ kit. Data are shown as mean ± SD.*P < 0.05, ** *P* < 0.01, compared with the control.

We evaluated the effect of 11S glycinin on the viability of IPEC-J2 cells using the CCK8 assay. 11S glycinin exhibited a strong inhibitory effect on the proliferation of IPEC-J2 cells in a dose-dependent manner (*P* < 0.05 or *P* < 0.01, Figure 1A). To further detect the inhibition of 11S glycinin on the proliferation of IPEC-J2 cells, LIVE/DEAD™ staining was used. The quantity of dead cells increased with the raise of 11S glycine concentration, which was consistent with the results of CCK8 analysis (Figure 1B). All of the aforementioned results demonstrated that 11S glycine could suppress IPEC-J2 cells.

### 11S Glycinin Induced ROS Generation and Decreased MMP in IPEC-J2 Cells

**Figure 2:**
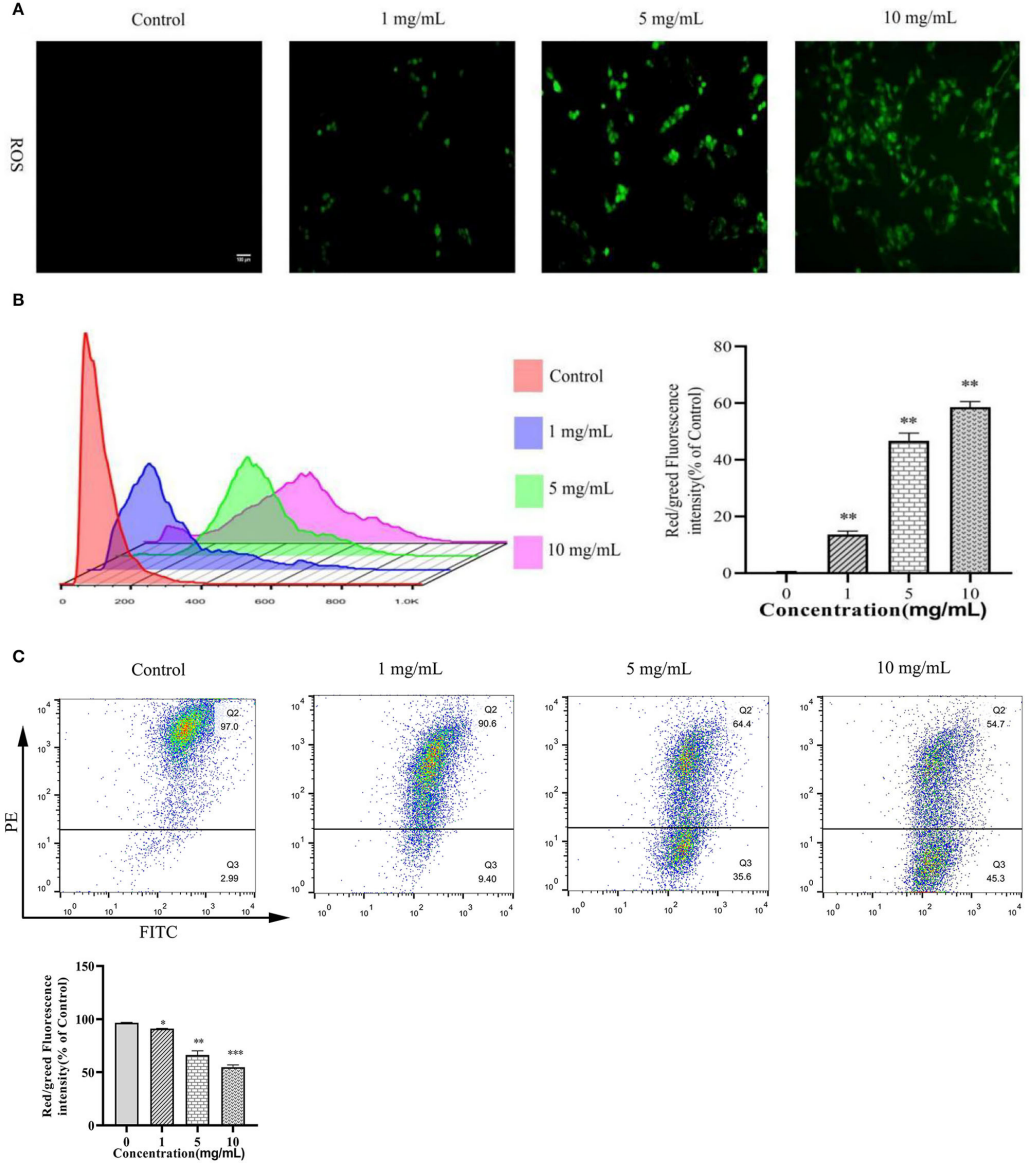
11S glycinin induced ROS generation and reduced MMP in IPEC-J2 cells. **(A)** IPEC-J2 cells were stimulated with 0, 1, 5, and 10 mg·ml^−1^ 11S glycinin for 24 h, and ROS were detected using DCFH–DA staining. **(B)** The red/green fluorescence intensity was measured using flow cytometry. **(C)** The mitochondrial membrane potential in IPEC-J2 cells were measured using the flow cytometry. Data are shown as mean ± SD. *P < 0.05, **P < 0.01, ***P < 0.001, compared with the control.

As shown in Figures 2A,B, ROS generation was significantly increased after 11S glycinin treatment (*P* < 0.01). As shown in Figure 2C, the mitochondrial membrane potential of IPEC-J2 cells which was treated with 11S glycinin was significantly reduced, compared with the control group (*P* < 0.05, *P* < 0.01 or *P* < 0.001).

### 11S Glycinin Induced DNA Damage and Increased the NLRP-3 Expression in IPEC-J2 Cells

**Figure 3:**
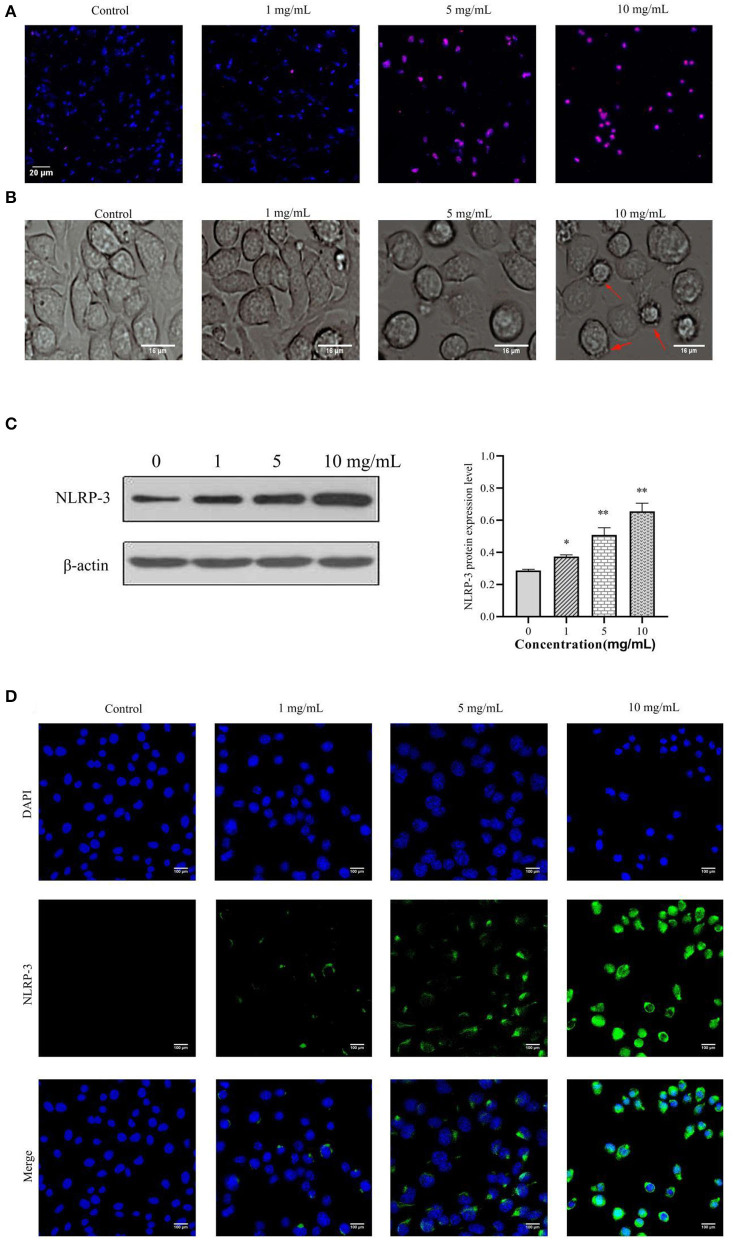
11S glycinin induced DNA damage and increased the NLRP-3 expression in IPEC-J2 cells. IPEC-J2 cells were treated with different concentration of 11S glycinin (0, 1, 5, or 10 mg·ml^−1^) for 24 h, respectively. **(A)** TUNEL shown the DNA damage of IPEC-J2 cells. **(B)** The morphology of IPEC-J2 cells after 11S glycinin treatment. **(C)** Western blotting presented the protein expression level of NLRP-3 in IPEC-J2 cells. **(D)** Immunofluorescence imaging of the NLRP-3 antibody in IPEC-J2 cells. Data are shown as mean ± SD. *P < 0.05, **P < 0.01, compared with the control group.

We evaluated whether 11S glycinin induced DNA damage in IPEC-J2 cells by TUNEL staining. As presented in Figure 3A, after treating the cells with 11S glycinin, the immunofluorescence intensity significantly increased, indicating that the number of positive cells increased significantly. According to the principle of TUNEL assay, the increase of positive cells evidenced that their DNA was fragmented. As revealed in Figure 3B, the morphology of IPEC-J2 cells in the low concentration (1 mg·ml^−1^) group was slightly damaged. In the middle concentration (5 mg·ml^−1^) group, the morphology of IPEC-J2 cells had reduced. While in the high concentration (10 mg·ml^−1^) group, we observed significant morphological changes, including cell swelling and the generation of large bubbles, which was highly redolent of pyrolysis. The NLRP-3 protein was assessed by the western blotting and immunofluorescence. As shown in Figures 3C,D, after 11S glycinin stimulation, the NLRP-3 protein expression (*P* < 0.05 or *P* < 0.01) and immunofluorescence signal were increased significantly.

### 11S Glycinin Induced Pyroptosis by Up-Regulating NLRP-3 in IPEC-J2 Cells

**Figure 4:**
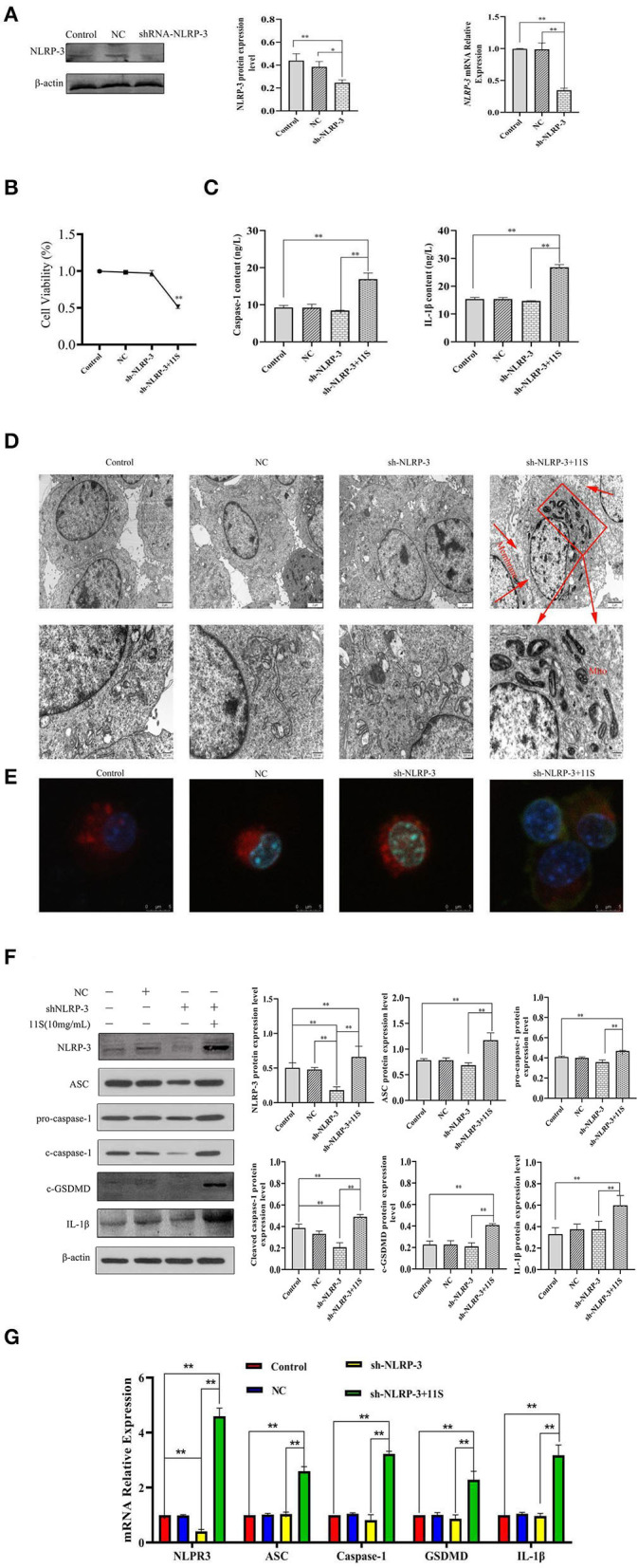
11S glycinin induced pyroptosis by up-regulating the silenced *NLRP-3* gene in IPEC-J2 cells. IPEC-J2 cells were transfected with designed sh-NLRP-3 lentivirus, then treated with 11S glycinin (10 mg·ml^−1^) for 24 h. **(A)** Western blotting and qRT-PCR verified the silencing effect of the target gene, *NLRP-3*. **(B)** The cell viability of IPEC-J2 cells was assessed using the CCK-8 assay, *n* = 4. **(C)** The content of caspase-1 and IL-1β determined using ELISA. **(D)** The ultrastructure of IPEC-J2 cells was observed *via* transmission of electron microscopy. **(E)** The fluorescent photographs of MMP in IPEC-J2 cells. **(F)** Western blotting indicated the expression level of NLRP-3, ASC, pro-caspase-1, cleave-caspase-1, c-GSDMD, and IL-1β in IPEC-J2 cells. **(G)** qRT–PCR results showed the mRNA expression of *NLRP-3, ASC, caspase-1, GSDMD*, and *IL-1*β in IPEC-J2 cells. Data are shown as mean ± SD. *P < 0.05, **P < 0.01, compared with the control group. Control (untreated); NC (negative control, transfected IPEC-J2 cells with empty vector lentivirus); sh-NLRP-3 (transfected IPEC-J2 cells with designed sh-NLRP-3 lentivirus); sh-NLRP-3+11S (transfected IPEC-J2 cells with designed sh-NLRP-3 lentivirus, then added 10 mg·ml^−1^ 11S glycinin and cultured for 24 h).

We transfected IPEC-J2 cells with the designed sh-NLRP-3 lentivirus and successfully silenced the *NLRP-3* (Figures 4A,B). As Figure 4C shows, transfection of the designed sh-NLRP-3 lentivirus did not affect the cell activity of IPEC-J2,but the cell activity of IPEC-J2 transfected with the designed sh-NLRP-3 lentivirus decreased significantly after stimulation with 10 mg·ml^−1^ 11S glycinin (*P* < 0.01**)**. As Figure 4D shows, we observed the typical pyroptosis morphology in sh-NLRP-3+11S group, including cell membrane rupture, cell swelling, and mitochondrial degeneration. The green fluorescent significant enhancement in sh-NLRP-3+11S group cells suggests that mitochondrial membrane potential was decreased (Figure 4E). As shown in Figures 4C,G, the cells of sh-NLRP-3+11S group showed a significant increase in protein level and mRNA expression of NLRP-3, apoptosis-related spot-like protein (ASC), cysteine-containing aspartate-specific proteases-1 (caspase-1), c-GSDMD, and IL-1β (*P* < 0.05 or *P* < 0.01). The contents of proinflammatory factor caspase-1 and IL-1β were assessed by ELISA. As shown in Figure 4F, the contents of caspase-1 and IL-1β in sh-NLRP-3+11S group were significantly increased than control group and NC group (*P* < 0.01).

### 11S Glycinin Up-Regulated NLRP-3 by Triggering ROS in IPEC-J2 Cells

**Figure 5:**
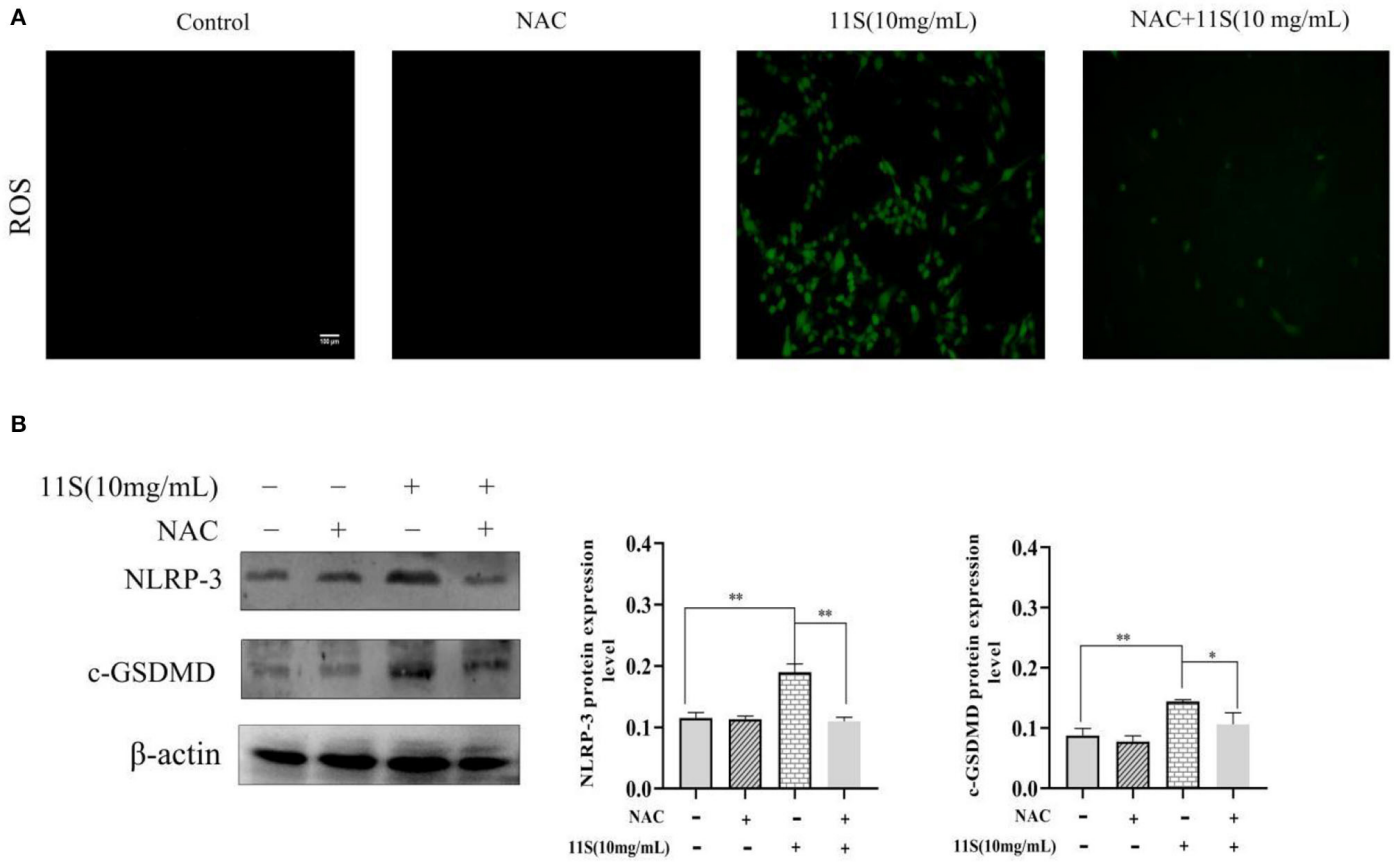
11S glycinin up-regulates NLRP-3-induced pyroptosis by triggering ROS in IPEC-J2 cells. **(A)** IPEC-J2 cells were treated with or without NAC (6 mM) for 1 h before a 24 h treatment of 11S glycinin (10 mg·ml^−1^). **(A)** ROS were detected using DCFH–DA staining. **(B)** Western blotting indicates the protein expression of NLRP-3 and c-GSDMD in IPEC-J2 cells. Data are shown as mean ± SD. *P < 0.05, **P < 0.01, compared with the control group.

As shown in Figure 5A, the ROS immunofluorescence signal of IPEC-J2 cells in 11S glycinin treatment group was significantly enhanced. Figure 5B showed a significant increase in the protein expression of NLRP-3 and GSDMD after incubation with 10 mg·ml^−1^ 11S glycinin at 24 h (*P* < 0.01). Treatment with the inhibitor (NAC) significantly weakened the ROS immunofluorescence signal, inhibited the up-regulated expression of NLRP-3 and GSDMD (*P* < 0.05 or *P* < 0.01).

## Discussion

Previous studies demonstrated that soybean antigen protein allergic can induce IPEC-J2 cell apoptosis (11), and can lead to inflammatory injury of the piglet small intestine (12). However, in the pathogenesis of acute and chronic enteritis, cell death has many forms, including apoptosis, cell scorching, iron death, necrosis, and autophagy. Although a recent study reported that common allergens can cause pyroptosis and lead to allergic diseases (19), the role of pyroptosis in allergy with 11S glycinin remains to be understood. Pyroptosis was a kind of death that was characterized by cell membrane rupture, which shares certain features with apoptosis, such as a decrease in mitochondrial membrane potential and DNA fragmentation (20). Our results demonstrated that 11S glycinin induced mitochondrial membrane potential decrease. We also demonstrated that 11S glycinin induced DNA damage in IPEC-J2 cells using the TUNEL assay; this contributes to pyrolysis. With the increase in the concentration of 11S glycinin, we observed distinct changes in morphology, including cellular swelling and the production of bubbles on IPEC-J2 cell membrane, which resemble the pyroptosis induced by the gasdermin D (GSDMD) (17, 21).

Recently, it has been shown that *NLRP-3* was an inflammatory compound widely existing in epithelial cells and played an important role in the occurrence and development of allergic diseases (22). We found that the expressions of *NLRP-3*, caspase-1, and IL-1β were significantly elevated in IPEC-J2 cells with increasing concentrations of 11S glycinin. The accumulation of NLRP3 inflammasome is considered to be an important activator of pyroptosis. Mature NLRP3 induces adapter ASC polymerization and ASC spot assembly (23, 24). ASC spots recruit and activate caspase-1, which then triggers the cleavage of GSDMD family proteins and promotes the release of interleukin-1β (IL-1β), as well as pyroptosis (25, 26). Therefore, we speculated that 11S glycinin may induce pyroptosis of IPEC-J2 cells by activating *NLRP-3*.

Few studies have shown the role of pyroptosis in 11S glycinin allergy. In order to fully prove our conjecture, we used the designed sh-NLRP-3 lentivirus to transfect IPEC-J2 cells and successfully silenced the target gene, *NLRP-3*. The *NLRP-3* gene silenced IPEC-J2 cells that were incubated with 11S glycinin for 24 h. Our results demonstrated that 11S glycinin significantly up-regulated mRNA and protein levels in IPEC-J2 cells of the NLRP-3 and other signaling molecules downstream, such as ASC, caspase-1, and IL-1β. More importantly, the mRNA of GSDMD and the protein levels of c-GSDMD were significantly increased as well. The secretion of IL-1β increased, which is common in pyroptosis. A recent study showed that GSDMD was an executor of pyroptosis and was required for the secretion of IL-1β (27). Furthermore, 11S could reduce the mitochondrial membrane potential in IPEC-J2 cells when the *NLPR-3* gene was silenced. Under the transmission electron microscope, the IPEC-J2 mitochondria were degenerated, the mitochondrial cristae disappeared, and the cell membrane was damaged, which were the main morphological features of pyroptosis. Our results suggested that 11S glycinin up-regulated *NLRP-3* expression, activated caspase-1, cleaved GSDMD, induced pyroptosis, and released inflammatory factors, which may be responsible for amplifying the inflammatory response. These results have not been reported earlier.

It is widely accepted that inflammation and oxidative stress usually occur simultaneously. ROS is an important cellular signal product responsible for oxidative stress (28), and is related to intestinal tissue injury and intestinal cell death (29, 30). ROS could promote the cleavage of GSDMD by activating NLRP-3, thereby inducing inflammatory processes (31). In this study, we verified that ROS production increased in a dose-dependent manner after 11S soybean globulin treated IPEC-J2 cells. The addition of NAC, a specific inhibitor of ROS, significantly weakened the fluorescence expression of ROS. More importantly, we also found that NAC significantly reduced the expression of NLRP-3 and c-GSDMD. Therefore, we believed that 11S glycinin induced IPEC-J2 cell pyroptosis, which was related to ROS and NLRP-3 in IPEC-J2 cells.

## Conclusion

In conclusion, our results demonstrated that 11S glycinin induced cell membrane rupture, DNA damage, mitochondrial membrane potential decrease, and ROS generation in IPEC-J2 cells. Furthermore, 11S glycinin up-regulated the expression of NLRP-3, activated caspase-1, cleaved GSDMD, and promoted the secretion of inflammatory cytokine IL-1β. Reducing ROS production could inhibit the expression of NLRP-3 and cleaved GSDMD. Taken together, our findings demonstrated that 11S glycinin up-regulated NLRP-3-mediated pyroptosis by triggering ROS in IPEC-J2 cells. This study provided new ideas for the prevention and treatment of the allergic diseases, which was induced by 11S glycinin. Allergic reaction in the organism is a very complex process involving multiple molecular pathways. However, our results were based on *in vitro* experiments, only revealing one of the possible mechanisms. Therefore, in the subsequent studies, weaned piglets will be used to construct animal models, and many prospective clinical trials will be conducted to reveal the mechanism of soybean antigenic protein-induced allergy.

## Data Availability Statement

The original contributions presented in the study are included in the article/supplementary material, further inquiries can be directed to the corresponding author.

## Author Contributions

JW and CP conceived and designed this experiment. JW coordinated financial and technical support. LW and ZS performed experiments and wrote the manuscript. WX and HD collect the experimental samples. JW, YL, SF, XW, and CZ reviewed the manuscript. All authors read and approved the final manuscript.

## Funding

This research was supported by the National Natural Science Foundation of China (No. 31972750).

## Conflict of Interest

The authors declare that the research was conducted in the absence of any commercial or financial relationships that could be construed as a potential conflict of interest.

## Publisher's Note

All claims expressed in this article are solely those of the authors and do not necessarily represent those of their affiliated organizations, or those of the publisher, the editors and the reviewers. Any product that may be evaluated in this article, or claim that may be made by its manufacturer, is not guaranteed or endorsed by the publisher.

## References

[1] Friedman M Brandon DL. Nutritional and health benefits of soy proteins. J Agric Food Chem. (2001) 49:1069–86. 10.1021/jf0009246 11312815

[2] Green B Cummings K Rittenour W Hettick J Bledsoe T Blachere F. Occupational sensitization to soy allergens in workers at a processing facility. Clin Exp Allergy NLM. (2011) 41:1022–30. 10.1111/j.1365-2222.2011.03756.x 21545549

[3] Gagnon C Poysa V Cober ER Gleddie S. Soybean allergens affecting North American patients identified by 2D gels and mass spectrometry. Food Anal Methods. (2010) 3:363–74. 10.1007/s12161-009-9090-3

[4] Murakami H Ogawa T Takafuta A Yano E Zaima N Moriyama T. Identification of the 7S and 11S globulins as percutaneously sensitizing soybean allergens as demonstrated through epidermal application of crude soybean extract. Biosci Biotechnol Biochem. (2018) 82:1408–16. 10.1080/09168451.2018.1460573 29629624

[5] Zheng S Qin G Chen J Zhang F. Acidic polypeptides A 1a, A 3, and A 4 of Gly m 6 (glycinin) are allergenic for piglets. Vet Immunol Immunopathol. (2018) 202:147–52. 10.1016/j.vetimm.2018.06.003 30078589

[6] Taliercio E Kim SW. Epitopes from two soybean glycinin subunits are antigenic in pigs. J Sci Food Agric. (2013) 93:2927–32. 10.1002/jsfa.6113 23426933

[7] Sun H Liu X Wang Y Liu J Feng J. Soybean glycinin- and β-conglycinin-induced intestinal immune responses in a murine model of allergy. Food Agr Immunol. (2013) 24:357–69. 10.1080/09540105.2012.704507

[8] Qiao S Li D Jiang J Zhou H Thacker PA. Effects of moist extruded full-fat soybeans on gut morphology and mucosal cell turnover time of weanling pigs. Asian Austral J Anim. (2003) 16:1. 10.5713/ajas.2003.63

[9] Zhao Y Qin G Sun Z Zhang B Wang T. Effects of glycinin and β-conglycinin on enterocyte apoptosis, proliferation and migration of piglets. Food Agr Immunol. (2010) 21:209–18. 10.1080/09540101003596644

[10] Peng C Sun Z Wang L Shu Y Wu J. Soybean antigen protein induces caspase-3/mitochondrion-regulated apoptosis in IPEC-J2 cells. Food Agr Immunol. (2020) 31:100–19. 10.1080/09540105.2019.1702926

[11] Peng C Ding X Zhu L He M Shu Y Zhang Y. β-conglycinin-induced intestinal porcine epithelial cell damage via the nuclear factor κB/mitogen-activated protein kinase signaling pathway. J Agric Food Chem. (2019) 67:9009–21. 10.1021/acs.jafc.9b02784 31319030

[12] Peng C Cao C He M Shu Y Tang X Wang Y. Soybean glycinin and β-conglycinin-induced intestinal damage in piglets via the p38/JNK/NF-κB signaling pathway. J Agric Food Chem. (2018) 66:9534–41. 10.1021/acs.jafc.8b03641 30139257

[13] Yu X Wang M Zhao H Cao Z. Targeting a novel hsa_circ_0000520/miR-556-5p/NLRP3 pathway-mediated cell pyroptosis and inflammation attenuates ovalbumin (OVA)-induced allergic rhinitis (AR) in mice models. Inflamm Res. (2021) 70:9809. 10.1007/s00011-021-01472-z 34028600

[14] Zasłona Z Flis E Wilk M Carroll G O'Neill L. Caspase-11 promotes allergic airway inflammation. Nat Commun. (2020) 11:1. 10.1038/s41467-020-14945-2 32103022 PMC7044193

[15] Cookson BT Brennan MA. Pro-inflammatory programmed cell death. Trends Microbiol. (2001) 9:113–4. 10.1016/S0966-842X(00)01936-3 11303500

[16] Man S Karki R Kanneganti TD. Molecular mechanisms and functions of pyroptosis, inflammatory caspases and inflammasomes in infectious diseases. Immunol Rev. (2017) 277:61–75. 10.1111/imr.12534 28462526 PMC5416822

[17] Shi J Zhao Y Wang K Shi X Wang Y Huang H. Cleavage of GSDMD by inflammatory caspases determines pyroptotic cell death. Nature. (2015) 526:660–5. 10.1038/nature15514 26375003

[18] Yang R Yu H Chen J Zhu J Zhang Q. Limonin attenuates LPS-induced hepatotoxicity by inhibiting pyroptosis via NLRP3/gasdermin D signaling pathway. J Agric Food Chem. (2021) 69:982–91. 10.1021/acs.jafc.0c06775 33427450

[19] Tsai Y Chiang K Hung J Chang W Lin H Shieh JM. Der f1 induces pyroptosis in human bronchial epithelia via the NLRP3 inflammasome. Int J Mol Med. (2017) 41:757–64. 10.3892/ijmm.2017.3310 29207030 PMC5752164

[20] Lammert CR Frost EL Bellinger CE Bolte AC Lukens JR. AIM2 inflammasome surveillance of DNA damage shapes neurodevelopment. Nature. 580:647–52. 10.1038/s41586-020-2174-3 32350463 PMC7788527

[21] Xiao J Wang C Yao J Alippe Y Xu C Kress D. Gasdermin D mediates the pathogenesis of neonatal-onset multisystem inflammatory disease in mice. PLoS Biol. (2018) 16:e3000047. 10.1371/journal.pbio.3000047 30388107 PMC6235378

[22] Allen IC Jania CM Wilson JE Tekeppe EM Hua X Brickey WJ. Analysis of NLRP3 in the development of allergic airway disease in mice. J Immunol. (2012) 188:2884–93. 10.4049/jimmunol.1102488 22323538 PMC3294123

[23] Jin C Flavell RA. Molecular mechanism of NLRP3 inflammasome activation. J Clin Immunol. (2010) 30:628–31. 10.1007/s10875-010-9440-3 20589420

[24] Schroder K Tschopp J. The inflammasomes. Cell. (2010) 140:821–32. 10.1016/j.cell.2010.01.040 20303873

[25] Bergsbaken T Fink S Cookson BT. Pyroptosis: host cell death and inflammation. Nat Rev Microbiol. (2009) 7:99–109. 10.1038/nrmicro2070 19148178 PMC2910423

[26] Keller M Rüegg A Werner S Beer HD Active caspase-1 is a regulator of unconventional protein secretion. Cell. (2008) 132:818–31. 10.1016/j.cell.2007.12.040 18329368

[27] He W Wan H Hu L Chen P Wang X Huang Z. Gasdermin D is an executor of pyroptosis and required for interleukin-1β secretion. Cell Res. (2015) 25:1285–98. 10.1038/cr.2015.139 26611636 PMC4670995

[28] Yang S Han Y He J Yang M Zhang W Zhan M. Mitochondria targeted peptide SS-31 prevent on cisplatin-induced acute kidney injury via regulating mitochondrial ROS-NLRP3 pathway. Biomed Pharmacother. (2020) 130:110521. 10.1016/j.biopha.2020.110521 32717631

[29] Abbasi-Oshaghi E Mirzaei F Pourjafar M. NLRP3 inflammasome, oxidative stress, and apoptosis induced in the intestine and liver of rats treated with titanium dioxide nanoparticles: *in vivo* and *in vitro* study. Int J Nanomedicine. (2019) 14:1919–36. 10.2147/IJN.S192382 30936694 PMC6421874

[30] Li L Tan H Zou Z Gong J Zhou J Peng N. North American hyperthermia group preventing necroptosis by scavenging ROS production alleviates heat stress-induced intestinal injury. Int J Hyperthermia. (2020) 37:517–30. 10.1080/02656736.2020.1763483 32423248

[31] Zhang R Chen J Mao L Guo Y Hao Y Deng Y. Nobiletin triggers reactive oxygen species-mediated pyroptosis through regulating autophagy in ovarian cancer cells. J Agric Food Chem. (2020) 68:1326–36. 10.1021/acs.jafc.9b07908 31955565

